# Earliest evidence of granivory from China (Shanxi Formation) points to seeds as a food source and nursing habitat for insects in the earliest Permian humid tropical forests of Cathaysia

**DOI:** 10.1371/journal.pone.0311737

**Published:** 2024-10-14

**Authors:** Artai A. Santos, Torsten Wappler, Stephen McLoughlin

**Affiliations:** 1 Department of Paleobiology, Swedish Museum of Natural History, Stockholm, Sweden; 2 Natural History Department, Hessian State Museum, Darmstadt, Germany; 3 Department of Palaeontology, Institute of Geosciences, Rheinische Friedrich-Wilhelms-Universität Bonn, Bonn, Germany; CNRS-University of Lille, FRANCE

## Abstract

Three types of plant-insect interactions are identified on seeds from the lower Permian (Asselian) Shanxi and lower Shihhotse formations of the Taiyuan district, North China. This enhances the relatively meagre fossil record of seed predation in global late Paleozoic floras, adding the earliest record of granivory from Cathaysia. The dispersed seeds cannot be attributed with confidence to any particular plant group, but associated fossil leaves belong to a broad spectrum of plants, including Medullosales, Cycadales, Noeggerathiales, Gigantopteridales, Cordaitales, and Voltziales. Among 85 analysed seeds, six showed evidence of predation, referable to three damage types: DT074 and two new damage types that will be added to the forthcoming version of the fossil damage guide (DT274, DT430). These damage features indicate novel strategies of seed exploitation in the earliest Permian of China. The causal agents of the seed herbivory are difficult to resolve with certainty, but possible culprits include representatives of Palaeodictyopteroidea, although we cannot exclude other groups, such as Dictyoptera, Odonatoptera, Archaeorthoptera, Hemipteroidea or early holometabolan insects. The presence of damage features, together with a range of probable defensive structures (hairs, spines, apical horns, and thick integuments), suggests that an active arms race involving insects and plant reproductive structures was already well established by the early Permian.

## 1. Introduction

Seed predation (granivory) is a distinctive category of herbivory (a functional feeding group: FFG) that strongly influences seed viability, seedling establishment, plant abundance, diversity and coexistence [1–4] with deep consequences for the structure and maintenance of terrestrial ecosystems. This distinctive FFG also has economic implications, since many extant granivores are important crop pests affecting seed production and viability [5, 6]. The major seed-feeding organisms in extant ecosystems are invertebrates, vertebrates and fungi [2, 7]. More specifically, insects are the most important granivores in almost all terrestrial ecosystems [2, 4]. The pattern of seed predation by insects in modern ecosystems is highly structured as a consequence of co-evolution with plants at the chemical, anatomical, spatial and temporal levels [8]. Several examples of complex and highly specific adaptations have been developed by insects for seed predation. In turn, plants have generated complex physical and chemical defences against seed-predation [9–13].

Seeds play a crucial role in the life cycle of spermatophytes. They are generally small, remarkably nutritious and less abundant than other plant organs. The high nutritional value of seeds is attributed to the presence of not only the mature sporophyte embryo but also substantial energy-rich tissues (nucellus or endosperm), providing resources for seedling growth. Although granivory can eventually be beneficial for dispersal or development of some seeds [14, 15], it is detrimental to many others, rendering the seed nonviable for germination. This damage may occur directly to the embryo or to the tissues necessary for its development. Additionally, damage can occur indirectly by introducing fungi and other pathogenic or opportunistic microorganisms that lead to ovule or seed abortion [15, 16].

In the fossil record, seed predation is considered one of the 12 Functional Feeding Groups (sensu Labandeira and Wappler [17]). This category of interaction incorporates any general arthropod damage on fossil seeds. In this sense, it can encompass multiple feeding activities apart from direct seed-feeding behaviour. For example, damage types attributed to seed predation are variably produced by oviposition, chewing, piercing-and-sucking, boring, or mining, hence, different strategies of seed consumption were adopted by various insects in the past but are here grouped under the broad category of seed predation. The origin of granivory remains obscure, but descriptions of the earliest bona-fide evidence of seed-predation in the fossil record come from the late Paleozoic of Laurussia and Gondwana [18]. Thus far, no evidence of Paleozoic seed-predation has been reported from Cathaysian terrains (North China and South China blocks) until this study.

Evidence of seed predation in the Paleozoic is very sparse (Table 1). Laurussian fossil assemblages have been investigated more intensively for granivory than other regions, with identification of examples on upper Carboniferous (Pennsylvanian) and lower Permian (Cisuralian) radiospermic seeds of Medullosales, and platyspermic seeds affiliated with Cordaitales [19–24]. Records from Gondwanan floras are even less common and confined to early Permian seeds of probable cordaitalean and glossopteridalean affinity from Brazil, South Africa and possibly India [25, 26]. Consequently, much work remains to be undertaken to clarify the origin and early evolution of this functional feeding group in a palaeogeographical context.

**Table 1:** Selection of Paleozoic occurrences of seed-predation arranged in geochronological order.

Evidence of seed-predation from Paleozoic deposits					
Age	Deposits/ Area	Paleogeographical region	Seed	DTs	References
Late Permian (Lopingian)	Bletterbach Gorge, Dolomites region (northeastern Italy)	Laurussia	Platyspermic seed (unknown affinities)	DT074	Labandeira et al. [24]
Early Permian (Kungurian)	Colwell Creek Pond and Mitchell Creek Flats (Texas, USA)	Laurussia	Platyspermic seed (unknown affinities)	DT073, DT074, DT257 and DT124	Schachat et al. [23, 27]
Early Permian (Artinskian)	Hammanskraal Fm., Karoo Basin, (South Africa)	Gondwana	*Elatra leslii* (glossopterid) seeds	DT073	Anderson & Anderson [28]; McLoughlin et al. [26]
Early Permian (?late Asselian–middle Artinskian)	Rio Bonito Fm. (Paraná Basin, Brazil)	Gondwana	*Cordaicarpus* and *Samaropsis*-like (cordaitalean or glossopterid) seeds	DT74, DT399, DT400 and DT401	Barbosa dos Santos et al. [25]
Early Permian (Asselian)	Talchir Fm., Talchir Coalfield, (Odisha, India)	Gondwana	*Cordaicarpus* sp.	DT073?	Chandra & Singh [29]
**Early Permian (early Asselian)**	**Shanxi Fm. (Shanxi province, China)**	**Cathaysia (North China)**	***Carpolithus bullatus* and *Carpolithus* spp.**	**DT074 and two new DTs (D274 and DT430)**	**This work**
Carboniferous/ Permian?	Tunguska Basin (Russia)	Laurussia	Platyspermic seed (unknown affinities)	Unknown (punctures)	Sharov [19]; Shcherbakov [22]
Late Carboniferous (Pennsylvanian)	Yorkshire and elsewhere in the UK	Laurussia (Euramerican floral province)	*Trigonocarpus* sp. (Medullosales)	Unknown (central hole)	Scott and Taylor [21]
Late Carboniferous (Early Pennsylvanian)	Casevilla Fm. (Illinois, USA)	Laurussia (Euramerican floral province)	*Trigonocarpus parkinsoi* (Medullosales)	Unknown (central hole)	Jennings [20]

Determining the culprits of seed predation in the fossil record is difficult. In modern ecosystems, various species spanning six main insect orders (Coleoptera, Hemiptera, Thysanoptera, Diptera, Lepidoptera and Hymenoptera) have seed-predation habits [8, 12, 30, 31]. However, during the Paleozoic, some of these orders were notably scarce or absent, and other extinct insect groups with obscure ecological behaviours were much more common and diverse (e.g., Palaeodictyopteroidea, Odonatoptera, Archaeorthoptera). Consequently, the search for the culprits of Paleozoic seed-predation remains challenging and speculative.

Given its apparent scarcity in Paleozoic floras, a re-investigation of Halle’s [32] extensive collections of Permian plants from North China is warranted to clarify the origins of seed predation in the Cathaysian floral realm [32]. The main objectives of this work are to: (1) examine the Permian plant assemblages of the Shanxi and Lower Shihhotse formations to identify examples of seed-predation; (2) describe the various damage types of seed predation and identify any new DTs; (3) analyse the palaeoecological implications of these cases of seed predation; (4) infer putative culprits for the damage; and (5) discuss the paleogeographical and evolutionary implications of this evidence for early seed predation in Cathaysia.

## 2. Geological setting

**Fig 1:**
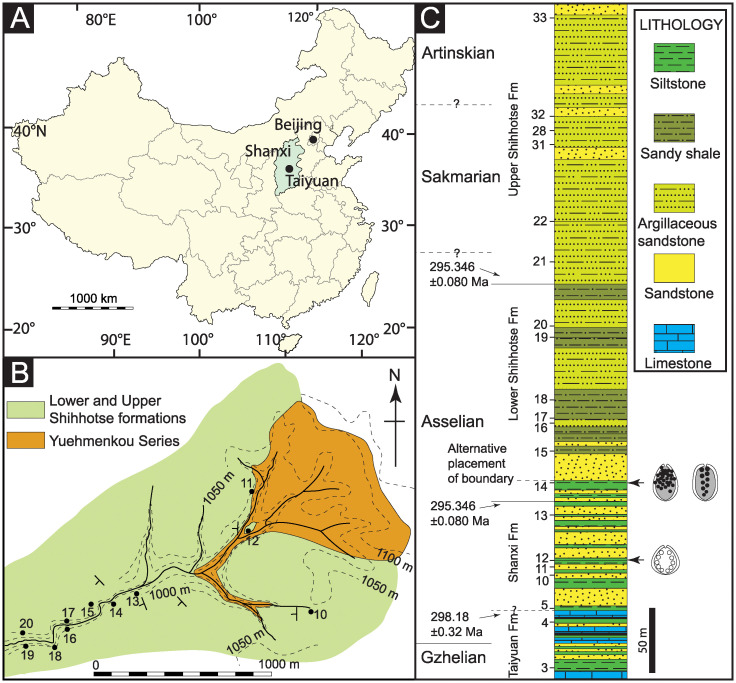
**A.** Geographical map showing the position of the studied site (basemap modified from China blank province map: Wikimedia commons https://commons.wikimedia.org/wiki/File:China_blank_province_map.svg); **B.** Geological map with exposures of the Lower and Upper Shihhotse formations (green), and the Yuemekou Series (orange) showing the location of Halle’s sampled beds; Modified after Halle [32]. **C.** Stratigraphic section of the studied site, showing the age (left), the beds recognised by Halle [32], the lithology from the East section of the Shihhotse Valley, and the stratigraphical position of the predated seeds (drawn on the right).

The upper Paleozoic strata of central Shanxi, China (Fig 1A and 1B), are divided into six principal rock units. In ascending stratigraphic order, these are the: Penchi (= Benxi) Formation (Pennsylvanian), Taiyuan Formation (Gzhelian–middle Asselian), Shanxi (= Shansi) Formation (middle to upper Asselian), Lower Shihhotse Formation (upper Asselian), Upper Shihhotse Formation (uppermost Asselian- lower Kungurian) and Sunjiagou (= Shiqianfeng) Formation (Lopingian) [33]. The succession appears conformable within the studied section, but regional correlations indicate local disconformities, diachronous transitions between the Penchi, Taiyuan, Shanxi and Lower Shhihotse formations across the North China Block, and a significant (c. 20-million-year) depositional hiatus between the Upper Shihhotse and Sunjiagou formations [33, 34].

The Penchi Formation consists of marine limestones, estuarine sandstones, mudstones, shales and paralic coal seams [35]. The succeeding Taiyuan Formation also incorporates black shales, coal seams, quartz sandstones and calcareous shales, intercalated with marine carbonates [36–38]. The overlying Shanxi Formation incorporates some equivalent lithologies but lacks limestones and marginal marine facies [39]. The succeeding Lower and Upper Shihhotse formations are dominated by sandstones, siltstones, claystones and sparse thin coals of alluvial plain settings, and these deposits yielded the majority of fossil plants described in Halle’s monograph of the Shanxi fossil flora [32]. The Sunjiagou Formation consists of red or brown claystones, sandstones and evaporites indicating a transition to dry continental depositional environments [40, 41]. The overall pattern of sedimentation is one of increasing continentality and shift to drier climates through the late Carboniferous and Permian [35].

The principal specimens studied herein derive from Halle’s fossil bed 12 (*Carpolithus bullatus*) and fossil bed 14 (cf. *Carpolithus* sp.) [32]. Halle [32] assigned bed 12 to the Lower Shihhotse Formation based on its stratigraphic position about 20 m above the Peitsakou Sandstone Member and the suggestion by Norin [42] that most of the Shanxi Formation was missing in the valley sections to the east of Taiyuan city. However, our reassessment of the correlations between sections to the west and east of Taiyuan outlined by Norin [42, 43] and Stevens et al. [35] suggests that Halle’s fossil bed 12 is within the Shanxi Formation (Fig 1C).

Halle’s fossil bed 14 is, similarly, difficult to place stratigraphically with certainty. Halle [32] indicated that bed 14 lies 80 m above the Peitsakou Sandstone Member in the Shihhotse Valley at Ch’en-chia-yü, placing this layer well within the Lower Shihhotse Formation. Stevens et al. [35] placed this bed only c. 15 m above the base of the Lower Shihhotse Formation. However, reconsideration of correlations between sections logged in the eastern and western hills of Taiyuan [42, 43], suggests that bed 14 lies near the top of an interval of mixed lithologies and immediately below a sandstone-dominated interval that probably correlates with the Lotopo (= Lotopoyao) ‘sandstone complex’. This would place bed 14 within the uppermost 2 m of the Shanxi Formation (Fig 1C).

Yang et al. [44] obtained LA-ICP-MS U-Pb dates from zircons in tuffs near the top of the Shanxi Formation in the Yongcheng Basin (southern North China Block) of 293.0±2.5 Ma (earliest Sakmarian). Wu et al. [34] recovered high-precision CA-ID-TIMS dates of 295.962±0.086 Ma and 295.346±0.080 Ma (mid-Asselian) from tuffs in the middle Shanxi Formation of the Palougou section at Baode, 175 km northwest of Taiyuan. Although somewhat younger ages have been recovered from the Shanxi Formation by analyses of detrital zircons, e.g., 262±2 Ma (Capitanian) at Xishan, Beijing Province [45], 281±4 Ma (Kungurian) at Pingliang in Gansu Province [46], and 287±3.7 Ma (Artinskian) in the Ordos Basin [47], these LA-ICP-MS ages are considered less reliable than high-precision CA-ID-TIMS dates derived from zircons within ash beds. Moreover, Zhu et al. [48] reported a youngest detrital zircon age of 299 Ma (Carboniferous-Permian boundary) from the basal sandstone of the Shanxi Formation in the Qingshui Basin. Given that LA-ICP-MS detrital zircon ages of 295 Ma have been recovered from the top of the underlying Taiyuan Formation, the balance of evidence suggests that the Shanxi Formation accumulated during the middle to late Asselian if this unit was deposited contemporaneously across the region [33].

Paleogeographic reconstructions of the North China Block suggest that, during deposition of the Shanxi Formation, the region around Taiyuan constituted a coastal plain with accumulation of significant coals and experiencing minor marine incursions. During accumulation of the succeeding Lower Shihhotse Formation, the area represented a coastal plain accumulating exclusively fluvial and lacustrine sands, silts and clays from adjacent upland areas [49]. The North China Block was located in tropical to subtropical northern latitudes (c. 10–30°N) during the Cisuralian [34, 50].

## 3. Material and methods

The collections of the Permian Shanxi province Permian flora housed in the Swedish Museum of Natural History (NRM) were examined for Permian seeds. In total, 85 seeds were recorded, belonging to 17 morphotypes classified in seven genera (see details in Table 2). The same taxonomic assignations of seeds employed by Halle [32] were used in this study.

**Table 2:** List of analysed fossil seeds from the Permian Shanxi flora with taxonomic assignments as provided by Halle [32] and including the number of specimens, damage types and apparent seed defences.

Fossil taxa	Number of specimens	Predated specimens	Seed defences
***Acanthocarpus lagenarius***	1 specimen	0	Thick coat
***Carpolithus bullatus***	2 specimens	2 specimens (DT430)	None
***Carpolithus* sp.**	4 specimens	4 specimens (DT074, DT274)	None
***Cordaicarpus* cf. *cordai***	1 specimen	0	Thick coat
***Cordaicarpus* sp. 1**	42 specimens	0	Thick coat
***Cordaicarpus* sp. 2**	1 specimen	0	Hairs/Spines
***Cornucarpus apertus***	1 specimen	0	Apical horns
***Cornucarpus carinatus***	1 specimen	0	Apical horns
***Cornucarpus incurvus***	2 specimens	0	Apical horns
***Cornucarpus patulus***	14 specimens	0	Apical horns
***Cornucarpus tenuicuspis***	1 specimen	0	Apical horns
***Cornucarpus minutus***	7 specimens	0	Apical horns
***Rhabdocarpus* sp.**	1 specimen	0	Thick coat
***Samaropsis sinensis***	2 specimens	0	None
***Samaropsis* sp.**	2 specimens	0	None
***Samaropsis taiyuanensis***	1 specimen	0	None
***Trigonocarpus norinii***	2 specimens	0	Thick coat

**Fig 2:**
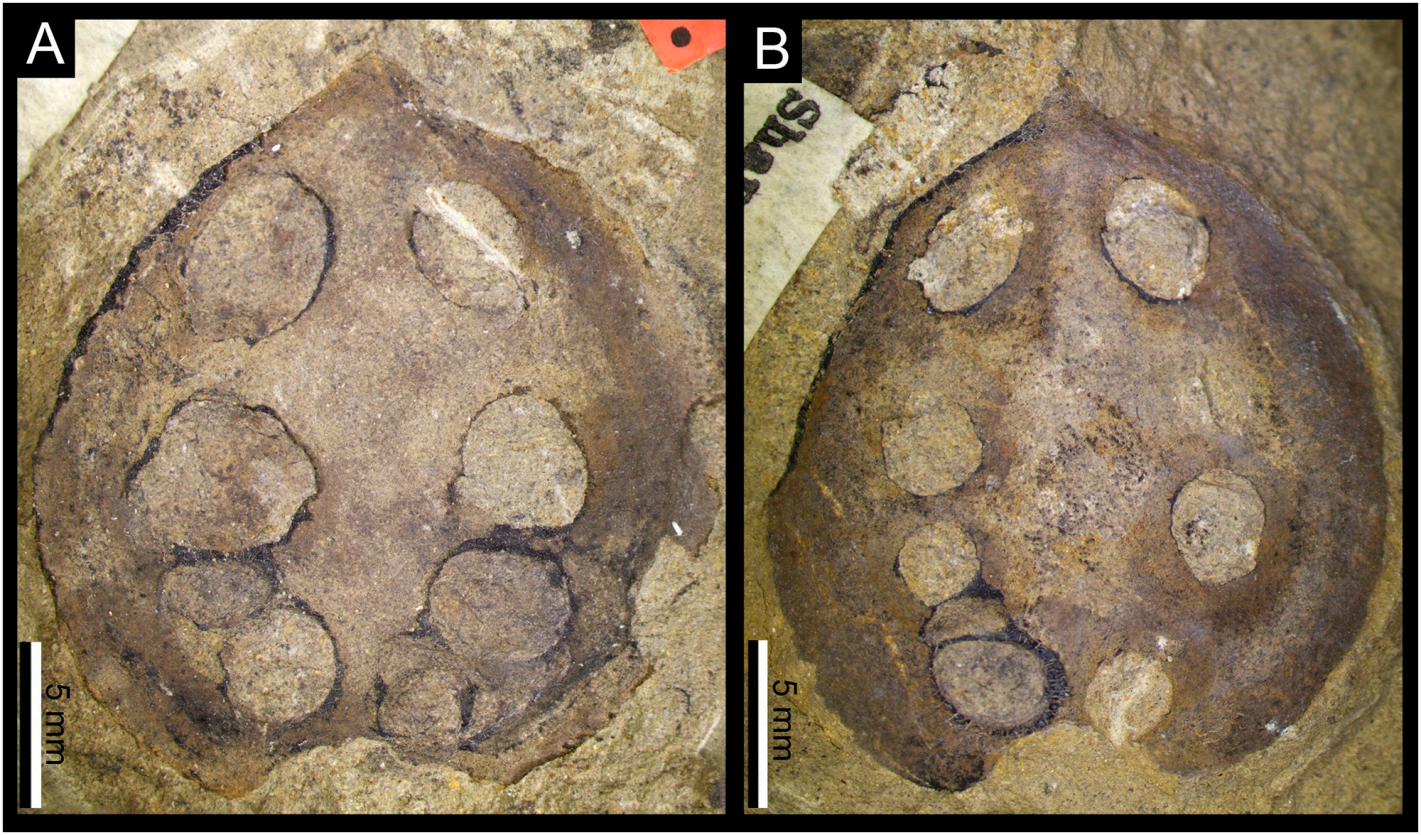
Illustrations of two specimens of *Carpolithus bullatus* showing the new Damage Type DT430; **A.** Holomorphotype for DT430 (NRM-S138324-01); **B.** Specimen NRM-S138325 with the same pattern of damage. Scale bars = 5 mm.

**Fig 3:**
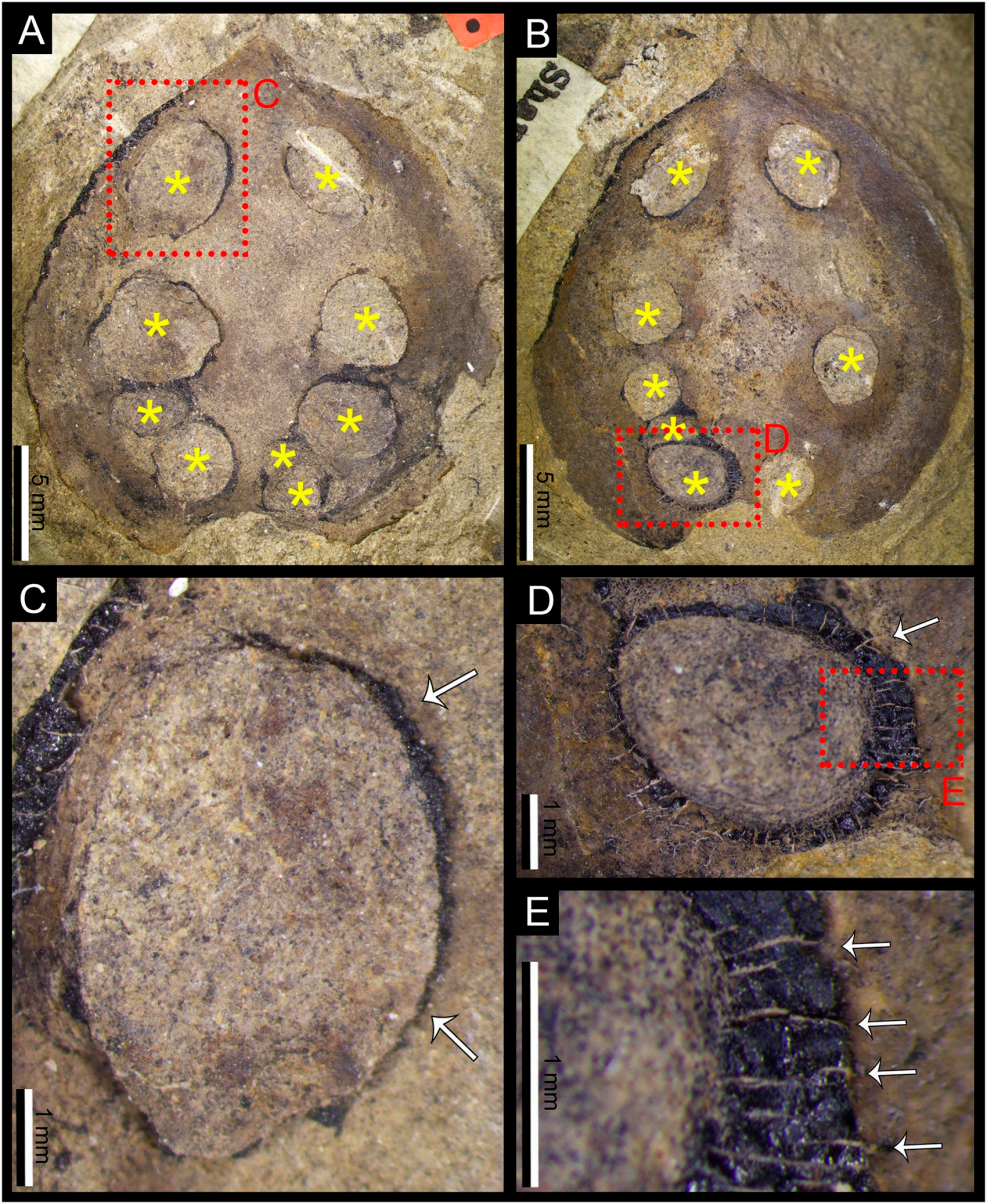
Details of the two specimens of *Carpolithus bullatus* affected by DT430; **A–B.** Specimens NRM-S138324-01 and NRM-S138325 with the distinctive pattern of DT430, showing the disposition of the multiple oviposition scars (yellow stars). Scale bars = 5 mm; **C.** Detail of A, showing the oviposition scar, white arrows indicate the dark reaction rim, suggesting reaction of the seed following damage. Scale bar = 1 mm; **D.** Detail of B showing one of the lenticular damage features on the seed, white arrow indicates the marked reaction rim surrounding this scar. Scale bar = 1 mm; **E.** Detail of the reaction rim of D; arrows show the transverse brown lines that probably represent diagenetic coal cleat mineral infillings. Scale bar = 1 mm.

**Fig 4:**
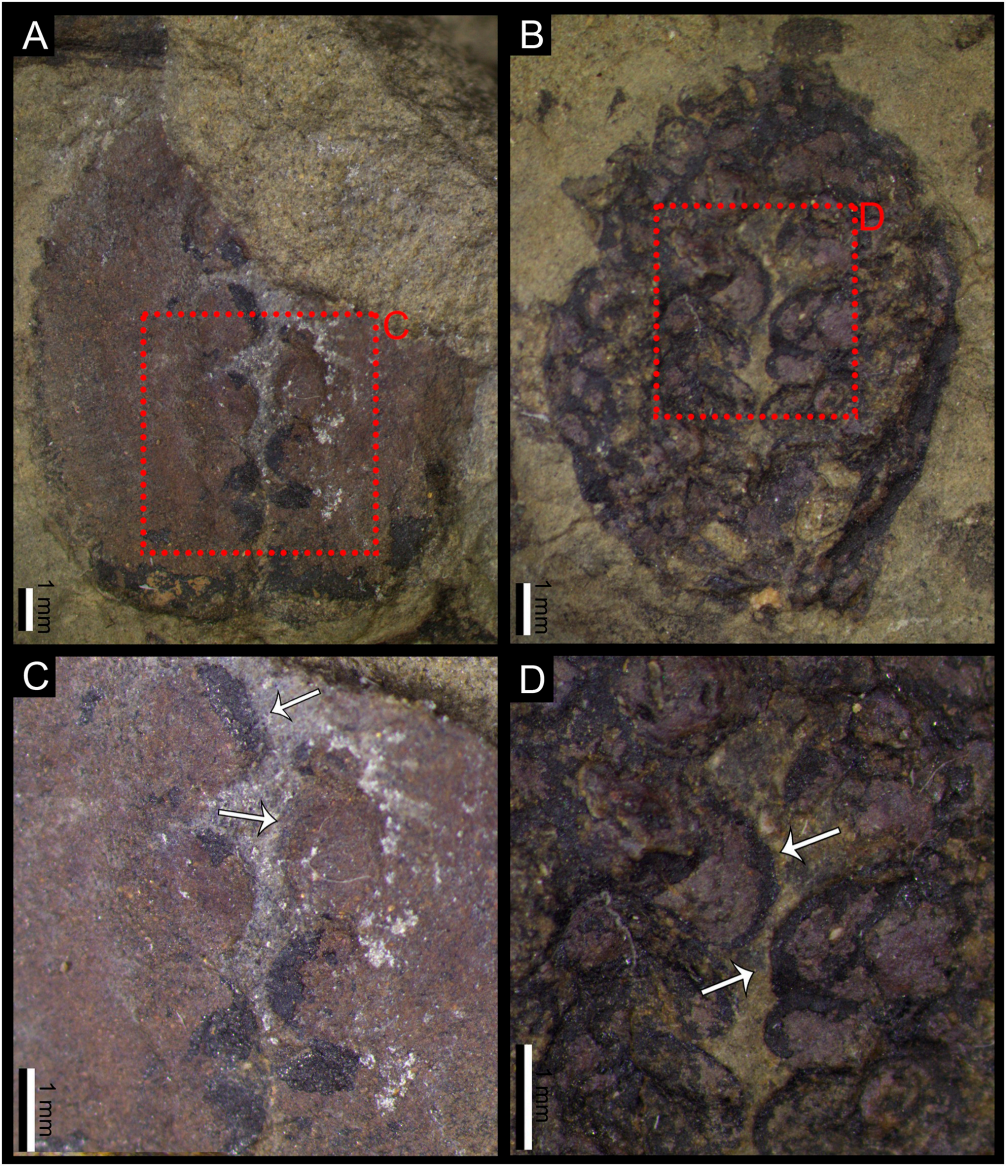
**A.** Specimen of *Carpolithus* (NRM-S138299-08) showing a new and distinctive category of seed predation damage, consisting in a “V”-shaped pattern of alternate oviposition scars, this damage represents a new DT (DT274). White arrow shows reaction rim surrounding one of the oviposition scars on the seed. Scale bar = 1 mm; **B.** Specimen of *Carpolithus* (NRM-S138299-02) showing damage features appear consistent with DT274. Scale bar = 1 mm; **C.** Detail of B (DT274), the alternate pattern of the damage marks is clear in this photo. The ovoid marks on the seed preserved as casts or mineral coatings (white arrows). Scale bar = 1 mm; **D.** Detail of B showing various scars, in some cases with dark reaction rims. Scale bar = 1 mm.

**Fig 5:**
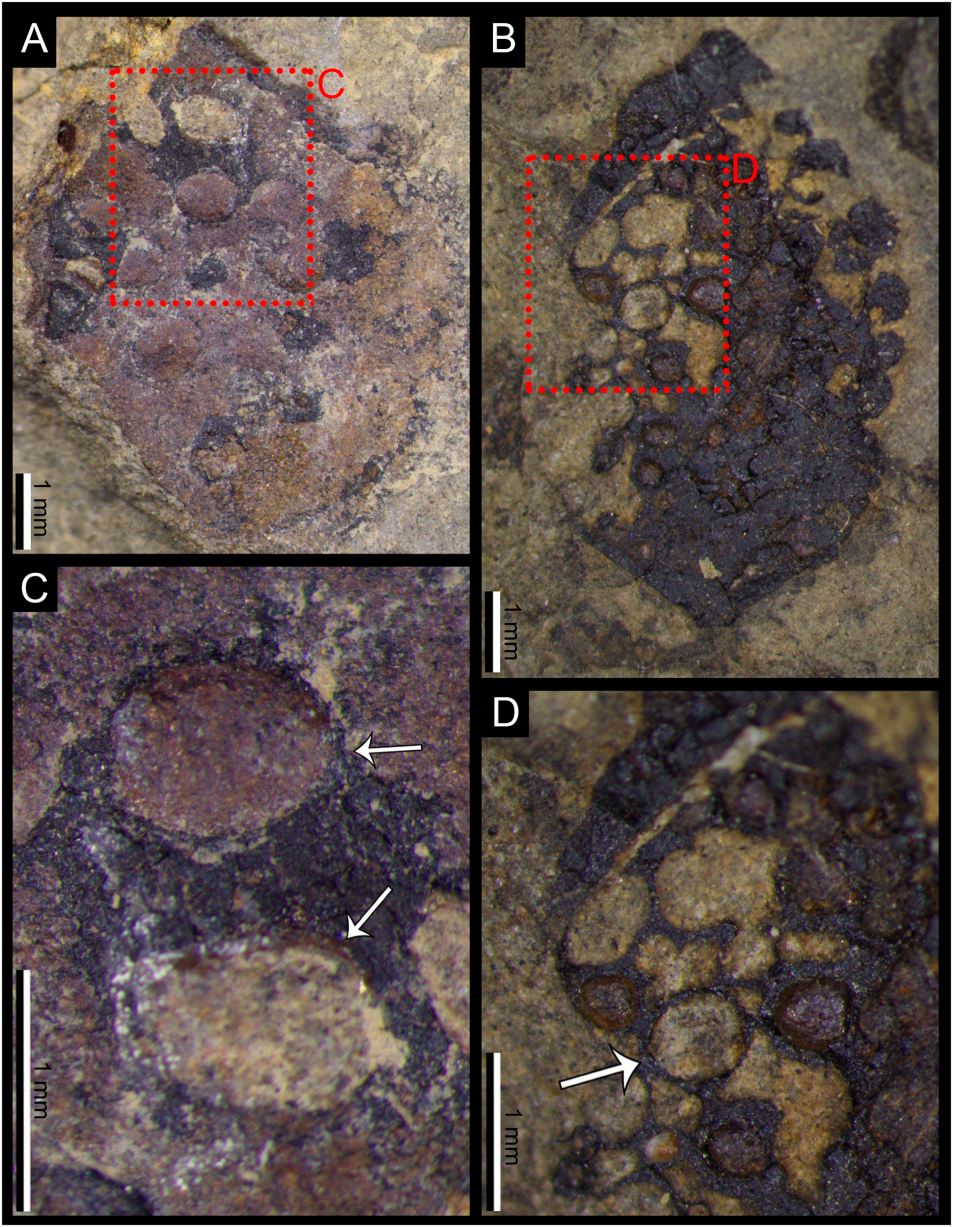
**A.** Specimen of *Carpolithus* sp. (NRM-S138299-03) showing evidence of seed predation (DT074). Note that some of the scars have a reaction rim around the punctate structures. Scale Bar = 1 mm; **B.** Specimen of *Carpolithus* sp. (NRM-S138299-01) showing damage consistent with DT074. Scale Bar = 1 mm; **C.** Detail of A (DT074), the ovoid marks on the seed are preserved as casts or mineral coatings (white arrows). Scale Bar = 1 mm; **D.** Detail of A, showing the ovoid marks on the seed (white arrow). Note that taphonomic processes have affected the preservation of the damage. Scale Bar = 1 mm.

**Fig 6:**
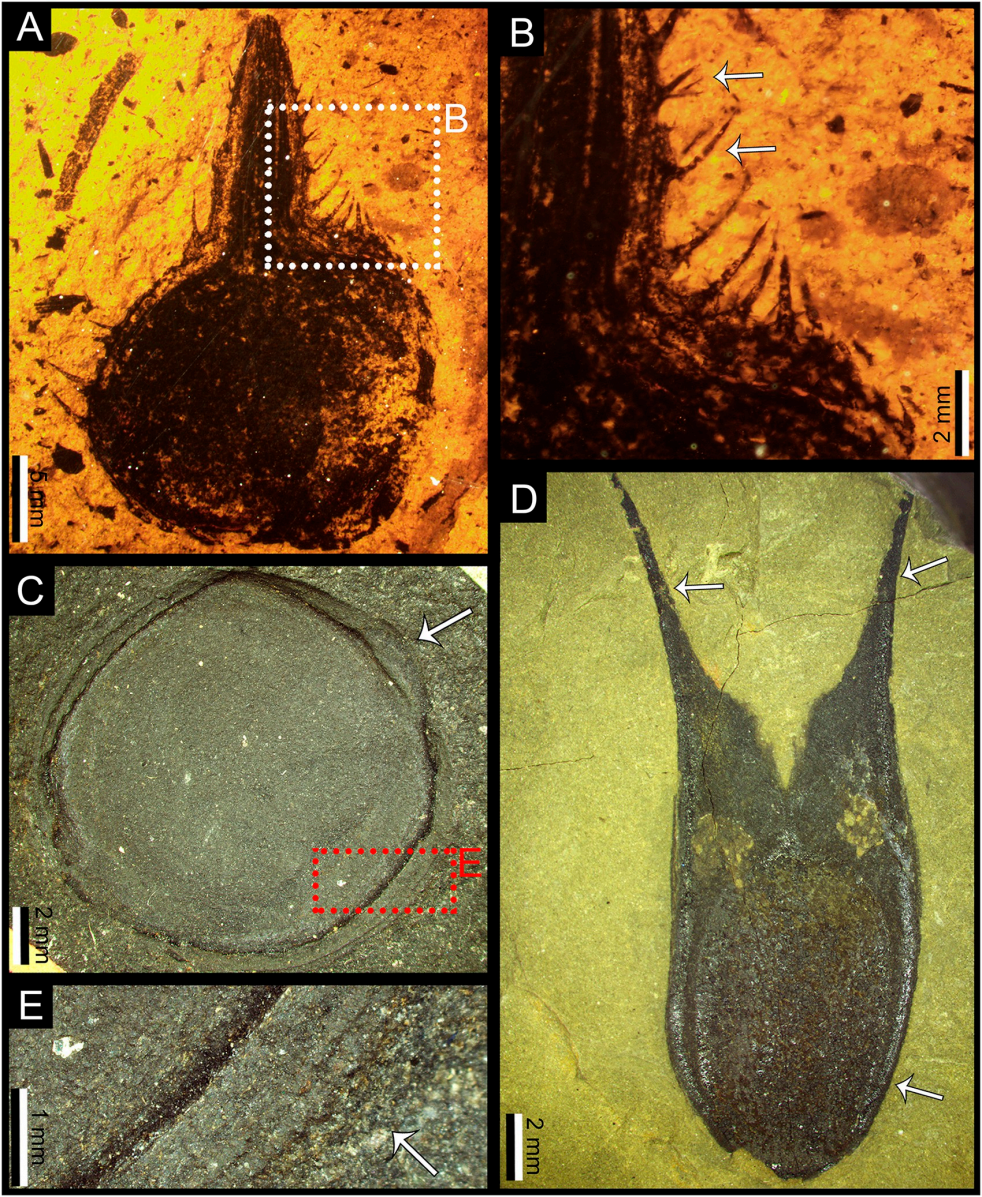
Various anti-herbivore defences on the Shanxi seeds; **A.** Specimen of *Cordaicarpus* sp. 2 (NRM-S138321a) with a thick integument, and various hairs/spines that cover the outer part of the seed, probably acting as deterrents to seed predation. Scale bar = 5 mm; **B.** Detail of the spines of *Cordaicarpus* sp. 2 (NRM-S138321a), white arrow shows presence of curved spines and putative hairs. Scale bar = 2 mm; **C.** Specimen of *Rhabdocarpus* sp. (NRM-S138318a) with a thick integument that might have acted as a protection against insect damage. Scale bar = 2 mm; **D.** Specimen of *Cornucarpus carinatus* (NRM-S138313) with two well-developed apical horns (white arrows), the function of these horns might be related to anemochorous dispersal, but might also have functioned as an anti-herbivore defence. Scale bar = 2 mm; **E.** Detail of C, showing the thick structure of the seed coat of *Rhabdocarpus* sp. Scale bar = 1 mm.

Seeds were carefully examined with a stereomicroscope to check for the presence of damage features. Six of the 85 seeds bore some form of damage (7%; Figs 2–5). The damage features were identified following the *Guide to Insect (and Other) Damage Types on Compressed Plant Fossils* [50] and subsequent (unpublished) addenda. In some cases, it was possible to assign the damage to an established Damage Type (DT) but, in other cases, new distinctive damage types were defined following the protocols of the guide. The 85 fossil seeds from the Shanxi flora were also carefully examined for possible plant defences against invertebrates, e.g., spines, hairs, horns, or thickened seed coat. Most of them (74) incorporated one or more structures that could be interpreted as seed defences against predators (see Table 2 and Fig 6). Only 11 seeds lacked apparent physical defences against insect damage. None of the six damaged seeds possessed any obvious defensive structures.

Photos of the illustrated material were taken with the camera of an iPhone 15 Pro with a stabilizer and different illuminations, detailed images were taken with an Olympus SZX10 stereomicroscope, equipped with an Olympus DP-71 digital camera in the laboratories of the NRM. All the studied material is housed and available in the palaeobotanical collections of the Department of Paleobiology in the NRM, Stockholm, Sweden.

## 4. Results

### 4.1. Description and diagnosis of damage features

#### 4.1.1. New damage type DT430 (Figs 2 and 3)

*FFG*. Seed Predation (seed predation is a composite FFG that combines DTs from other FFGs; this trace is attributed to oviposition in seeds).

*Damage type*. DT430.

*Feeding event occurrence data*. Pattern event occurrence.

*DT host specificity*. 3 (host specialist). The level of specificity is uncertain, but based on the two occurrences on specimens of the same seed species this damage appears to have been generated by a specialist. More occurrences of this DT are necessary to confirm this HS value.

*Locality*. Outcrop of Bed 12 (Halle [32]) in the Shihhotse Valley east of Taiyuan city, Shanxi Province, China (see Geological setting for details; Fig 1).

*Stratigraphy*. Shanxi Formation (see Geological setting for details; Fig 1).

*Age*. earliest Permian (early Asselian) about 296 Ma. (see Geological setting for details).

*Host plant*. *Carpolithus bullatus* Halle (details of the host plant are provided in the discussion).

*Inferred culprit*. Uncertain (see general discussion below).

*Specimens and repository*. DT430 was found in two specimens of *Carpolithus bullatus* (NRM-S138324-01 and NRM-S138325) preserved as impressions with minor remnants of coalified tissues and housed in the paleobotanical collections of the NRM. No other *Carpolithus* fossils from the collection hosted this type of damage.

*Holomorphotype of DT430*. NRM-S138324-01 (Figs 2A and 3A).

*Paramorphotype of DT430*. NRM-S138325 (Figs 2B and 3B).

*Diagnostic characters*.

1. Lenticular to ovoid pustules (on impression; = depressions or cavities on original seed) surrounded by a dark reaction rim of original seed tissue; arranged in two arcuate rows on each side of the seed midline.
2. Damage appears to have extended into the gametophytic or embryonic tissue of the ovule/seed.
3. Each lateral row consists of 3–5 oviposition scars of millimetric diameter.

*Detailed description of the damage*. Lenticular to ovoid (pustular) oviposition scars arranged in two arcuate rows that run approximately parallel to the lateral margins of the platyspermic ovule/seed (see description of the seed below). The scars penetrate into the interior of the testa, apparently in the fleshy tissues corresponding to the nucellus or embryo. Each row consists of three to five scars (4–5 in one seed and 3–5 in the other).

Individual oviposition scars differ slightly in size, ranging from 2.30 to 6.03 mm long and 1.2 to 5 mm wide, although these dimensions might have resulted from some taphonomic distortion. The average size of the scars is 3.84 mm long and 3.03 mm wide. The scars in each row are separated by 0.1–2.7 mm, with an average of 1.39 mm. The distance between each row of oviposition scars averages 3.55 mm, separating slightly in the central part of each row and converging towards the ends of each row.

The interactions appear to have a generally prominent black reaction rim, although this may simply represent entrapment of coalified tissues around the margins of the pustulose scars. This probable reaction tissue is variably expressed in the fossils, with widths of 0.1–0.65 mm (average = 0.3 mm). Some reaction rims incorporate thin radial or transverse streaks of white calcitic material that probably represents diagenetic mineral precipitation along coal cleat.

The oviposition scars are inserted deeply into the seed, passing through the seed coat and extending into the seed chamber, with depths ranging from 0.63 to 3.74 mm (average 2.32 mm).

*Description of the host plants*. The two examples of DT430 are represented on two specimens of ovate fossil seeds attributed to *Carpolithus bullatus* Halle: the holotype (NRM-S138324-01) and paratype (NRM-S138325). The two seeds have very similar structure and size (NRM-S138324 = 22.54 mm x 22.51 mm, and NRM-S138325 = 19.6 mm x 18.3 mm) with 2–4-mm-wide marginal wings. Further morphological details of these specimens were described by Halle [32].

*Discussion of the host plants*. Only two fossil seeds have ever been assigned to *Carpolithus bullatus* Halle, i.e., those illustrated here and described by Halle [32]. The diagnostic character of this taxon was stated to be the pustulose features flanking the margin, which Halle described as forming an “obscure structure” resembling “angiospermous fruits”, but which we interpret as insect damage. On this basis, we suggest that *Carpolithus bullatus* lacks characters differentiating it from other members of the genus, since the “knobs or swellings” described by the Halle do not seem to be anatomical features of the specimen.

We agree with Halle [32] that the seeds are attributable to *Carpolithus*. Note that *Carpolithus* Artis has nomenclatural priority over its synonym *Carpolithes* Schlotheim ex Brongniart [51, 52]. However, the botanical affinities of these seeds remain ambiguous. They are potentially affiliated with Cordaitales given that the foliage of this group is common in the same bed (Santos and McLoughlin, personal observation).

*Discussion of the damage*. DT430 consists of very distinctive oviposition scars penetrating the inner tissues of *Carpolithus bullatus* seeds. The robust reaction tissue surrounding the scars indicates a marked response of the plant tissue to the damage. The two available specimens of *Carpolithus bullatus* host the first and only examples of DT430 recorded so far. The seeds have an integument divided into a thin sclerotesta and an outer, narrowly winged sarcotesta. The inner part of the ovules/seeds, corresponding to the nucellus, may have been fleshy, and appears to host the oviposition scars. This inner nutritive tissue was probably the target for oviposition by the female insect. The scars are generally well separated, allowing the immature stages of these insects access to sufficient food and avoiding intraspecific competition.

The distinctive and regimented pattern of these oviposition scars in the same species of fossil seeds indicates a specialised strategy of oviposition and subsequent feeding behaviour. The penetration of the oviposition damage into the inner part of the seed might also have been a strategy to protect the eggs and early juvenile stages in addition to providing a ready food source. The rich food source might have facilitated a longer period of development of the insect inside the seed, thus, improving the chances of survival.

It is unlikely that the host plant received any advantage from this interaction, as the inner part of the seed, including the nucellus/embryo area seems to have been extensively damaged, likely making the seeds unviable.

We note that permineralised cardiocarpalean ovules of *Callospermarion undulatum* (Neely) Rothwell described from the lowermost Permian Taiyuan Formation of Shanxi have thick integuments with prominent cavities [53]. Even if these cavities were to be infilled with sediment, they are unlikely to correspond to the sediment-filled scars of *Carpolithus bullatus* given their smaller size, distinctive epithelial cells and regular spacing. Rather, they appear to be resinous secretory cavities although, intriguingly, several contain arthropod coprolites. Another anatomically preserved cardiocarpalean ovule, *Cardiocarpus dabiziae* Hilton et al., from the Taiyuan Formation bears prominent protuberances on the surface of the integument [37] that are similar in size to the scars on *C*. *bullatus*. However, given their consistent occurrence on seeds of *C*. *dabiziae* and their anatomical continuity with the integument, these structures are probably also some form of resinous secretory structures, possibly employed for seed defence, rather than insect eggs or pupal chambers.

#### 4.1.2. New damage type DT274 (Fig 4)

*FFG*. Seed Predation (oviposition scars in seeds).

*Damage type*. DT274.

*Feeding event occurrence data*. Pattern event occurrence.

*DT host specificity*. 3 (host specialist). The level of specificity is uncertain, more occurrences on this DT are necessary to confirm this HS value.

*Locality*. Outcrop of Bed 14 (Halle [32]) in the Shihhotse Valley east of Taiyuan city, Shanxi Province, China (see Geological setting for details; Fig 1).

*Stratigraphy*. Lower part of Lower Shihhotse Formation or uppermost Shanxi Formation (see Geological setting for details; Fig 1).

*Age*. earliest Permian (early Asselian) about 295 Ma. (see Geological setting for details).

*Host plant*. *Carpolithus* sp.

*Inferred culprit*. Unknown insect with well-developed ovipositor.

*Specimens and repository*. **DT274** was found in two specimens (NRM-S138299-02 and NRM-S138299-08) preserved as impressions with remnants of the coalified seed coat retained. No other seed type in the collection hosts this type of damage. The specimens are housed in the paleobotanical collection of the NRM.

*Holomorphotype of DT274*. NRM-S138299-08 (Fig 4A).

*Paramorphotypes of DT274*. NRM-S138299-02 (Fig 4B).

*Diagnostic characters*.

1. Lenticular to ovoid oviposition scars distributed in two parallel rows showing an alternate distribution, in decreasing size from the apical (micropylar) end of the seed.
2. Oviposition scars are distributed through the central part of the seed.
3. Each row is composed of four to five oviposition scars of millimetric size.

*Detailed description of the damage*. Two straight rows of scars slightly divergent, forming a close “V” shape through the central axis of the seed (angle of the “V” is about 5–8°). The “V” shape is slightly more open in NRM- NRM-S138299-08 than in NRM-S138299-02; individual scars alternating between each row. Reaction rim is weakly developed. Oviposition scars are lenticular to ovoid and decrease in size from the apical portion of the seed towards the base (chalaza). This pattern is slightly less well developed in NRM-S138299-02 than in specimen NRM-S138299-08. The central nucellus/embryo region of the ovule/seed is difficult to differentiate in both seeds, but the scars apparently penetrate these inner tissues.

Scars are 1.25–2.15 mm long and 0.95–1.78 mm wide (mean: 1.53 x 1.32 mm). The larger oviposition scars are c. 2.15 x 1.62 mm and the smaller are c. 1.14 x 0.67 mm. All scars are separated by less than 0.4 mm (typically between 0.31 mm and 0.05 mm; mean 0.17 mm). Separation between scars of opposing rows is 0.29–1 mm (mean: 0.42 mm), widening towards the seed apex. Oviposition scars embedded deep in the tissues of the seed; located 1.41–2.17 mm from the seed margin (mean: 1.6 mm).

*Description of the host plants*. The damage occurs in specimens NRM-S138299-02 and NRM-S138299-08. These seeds are mostly preserved whole, but an apical portion of NRM-S138299-08 is broken away. The seed NRM-S138299-08 is 13.1 mm long and 11.8 mm wide and NRM-S138299-02 is 10.2 mm long and 7.8 mm wide. The botanical affinities of the seeds are unresolved.

*Discussion of the damage*. DT274 consists of scars produced by endophytic oviposition in the inner tissues of gymnosperm seeds. The distinctive, slightly “V”-shaped arrangement and alternate positioning of the damage indicates that these features were made by a unique female with a strong ovipositor, owing to the scale of the scars. The difference in size between the apical and basal scars might be due to variation in the original angle of the ovipositor and the position of the seed. The alternate “V” shape of the damage array was probably produced by the movement of the ovipositor in a zig-zag pattern.

The penetration of the damage into the inner part of the seed might have been a strategy to increase the chances of larval survival. The inner tissues of the seed are clearly more nutritive for the younger phases of the insect that, after eclosion from the egg, would have found an abundance of food. In addition, eggs are a vulnerable phase of the insect life cycle, and their placement inside the seed, shielded by a robust integument may have afforded strong protection from predators. The spacing between the eggs was probably a means to reduce competition for food between individuals in the early stages of insect development.

The inner part of the seed (nucellus/embryo) appears to have been significantly damaged in this interaction, indicating that the host plant likely did not gain any advantage. The specificity of the damage suggests more than mere seed predation; it hints at a potential parasitic behaviour towards the seeds of these plants.

#### 4.1.3. Damage type DT074 (Fig 5)

*FFG*. Seed Predation (punctures in the seed).

*Damage type*. DT074.

*DT host specificity*. 2.

*Locality*. Outcrop of Bed 14 (Halle, [32]) in the Shihhotse Valley east of Taiyuan city, Shanxi Province, China (see Geological setting for details; Fig 1).

*Stratigraphy*. Lower part of Lower Shihhotse Formation or uppermost Shanxi Formation (see Geological setting for details; Fig 2).

*Age*. earliest Permian (early Asselian) about 295 Ma. (see Geological setting for details).

*Host plant*. *Carpolithus* sp.

*Inferred culprit*. Permothemistida (Diathemidae) or Hemiptera (Sternorrhyncha according to Barbosa dos Santos et al. [25]).

*Specimens*. NRM-S138299-01 (Fig 5B) and NRM-S138299-09 (Fig 5A).

*Description*. Small, circular to ovate punctures on various parts of the seed. Punctures 0.2–1 mm in diameter (with only two punctures exceeding 1 mm in NRM-S138299-09). These punctures resemble domal pustules (on impressions) corresponding to cratered pits or cavities (on the original seed) and each is flanked by a marked encircling rim. Scars occur primarily on the main body of the seed but are also present on the narrow-winged margins. The puncture pattern creates a disruptive appearance on the reticulate or granulate surface texture of the seed. These punctures penetrate both the outer and inner tissues of the host seed. The punctures are clustered, consisting of a variable number of damage marks that are closely spaced but never intersecting.

*Remarks*. This type of damage could fit the broad description of DT074 of Labandeira et al. [54] and subsequent addenda. Nevertheless, it has some differences, including the size of some punctures and the type of host plant (seed). We note that taphonomical processes could have contributed to some deformation of the seed and the damage features, thus slightly affecting their apparent size. On the other hand, Barbosa dos Santos et al. [25] noted this type of damage in *Cordaicarpus* sp. (possible cordaitalean or glossopterid seeds), whereas the Chinese specimens have less distinct features and are attributed to *Carpolithus* sp. with unresolved botanical affinities. The presence of this damage type in widely separated geographic regions during the Early Permian (China and Brazil) might indicate that this feeding strategy was broadly distributed in the early Permian forests, and seeds might have provided an important source of food for permothemistid Diathemidae or Sternorrhyncha insects across tropical and temperate regions. In addition, the high density of damage in these seeds (especially in NRM-S138299-01) seems to indicate that the host plant did not obtain any benefit from this interaction. The culprit insects appear to have had a marked preference for the central region of the seed, which was likely much more palatable and nutritive than the hard, coriaceous testa.

## 5. Discussion

### 5.1. The seed-producing plant, pre-dispersal and post-dispersal seed predation

Resolving whether the damage to fossil seeds occurred prior to seed dispersal is challenging. In the seed assemblages studied here, we noted some seeds occur attached to the parent plants (e.g., Halle [32], pl. 59, Figs 1–14), which are definitively in a pre-dispersed state. However, most of the seeds present in the Shanxi flora were found isolated from their parent plants and, in these cases, it is difficult to determine whether damage was due to pre-dispersal or post-dispersal attack. In some of the cases described above, apparent reaction rims around scars suggest that the damage occurred before the seeds had been shed.

The *Carpolithus bullatus* seeds examined in this study have not been found in physical connection to other plant organs. They are co-preserved in Halle’s [32] bed 12 with *Cordaites* sp., *Sphenophyllum oblongifolium*, *Sphenopteris* sp., *Chansitheca palaeosilvana*, *Pecopteris* spp., *Odontopteris subcrenulata*, *Taeniopteris latecostata*, *T*. *nystroemii*, *Taeniopteris* sp. cf. *T*. *schenki* and *Plagiozamites oblongifolius*. Of the seed-producing plants in this assemblage, *Odontopteris* foliage is probably affiliated with Medullosales [55], *Taeniopteris* leaves are of ambiguous affinities but possibly allied to early Cycadales [56] and *Plagiozamites* has probable affiliation to Noeggerathiales [57]. Affiliation of *Carpolithus bullatus* seeds to any one of these seed plants is not certain, but it usually occurs in close association with cordaitalean foliage.

The seeds assigned by Halle [32] to cf. *Carpolithes* sp. from fossil bed 14 are associated with foliage referred to *Annularites sinensis*, *Calamites suckowii*, *Sphenophyllum oblongifolium*, *S*. *thonii*, *Bowmanites laxus*, *Oligocarpia gothanii*, *Pecopteris* sp. cf. *P*. *arcuata*, *Cladophlebis nystroemii*, *Alethopteris ascendens*, *Emplectopteris triangularis*, *Protoblechnum wongii*, *Taeniopteris shansiensis*, *Gigantopteris whitei*, *Stigmaria ficoides*, *Cordaites* sp., unidentified coniferous remains and *Astrocupulites acuminatus*. The non-spore-producing plants are attributed to various seed-bearing groups, including Callistophytaceae (*Emplecopteris*: Seyfullah and Hilton [58]), Gigantopteridales (*Gigantopteris*), Cordaitales (*Cordaites*), conifers, possible Cycadales (*Taeniopteris*) and groups of unknown affinity (*Protoblechnum*). The few morphological features available on the impression fossils mean that cf. *Carpolithes* sp. cannot be allied confidently to any one of these plant groups; nor is there any clear correspondence in relative abundance that would suggest affinities with any one leaf type.

### 5.2. Evolutionary implications: The arms race between herbivores and seed defences

In the intricate dance of co-evolution, plants and herbivorous insects have engaged in a 400-Myr arms race shaped by the forces of natural selection [13, 59, 60]. Plants, aiming to safeguard themselves from herbivory, developed an array of anti-herbivore defences. Today, such defences are present in virtually all the terrestrial plant groups, and their constituent organs. Plant-defences may be manifest as chemical compounds, physical structures, such as hairs, horns or spines, or phenological changes [9–13].

The origin of anti-herbivore defences in plants can be traced back to the Carboniferous or Devonian [13, 59, 60]. However, studies of anti-herbivore defences in the Paleozoic are scarce, focusing predominantly on defences in vegetative organs, such as stems and leaves [13, 61, 62]. There is a notable dearth of published research on anti-herbivore defences in Paleozoic seeds. Here, we present evidence of seeds bearing various types of both insect-inflicted damage (Figs 2–5) and various anti-herbivore physical defences (Fig 6) in some of these ancient seeds. The identification of both complex damage patterns and physical defences indicates that the evolutionary arms race between plants and insects already involved seeds by the early Permian.

Anti-herbivory defences are expressed in the form of hairs, spines, thick seed coats and apical horns on several of the Shanxi seeds (Fig 6). Thorns have long been recognized as an effective anti-herbivore defence across various plant organs, although thorn-like apical horns on seeds, might also have served an aerodynamic dispersal function (anemochory) or, in the case of some Mesozoic seeds, have been employed for zoochory [63]. However, the presence of seeds with spines in the Permian (Fig 6A and 6B) unmistakably signifies deterrence of seed predation. Although some extant seeds may develop spines to adhere to animal fur or feathers, facilitating zoochory, the Permian lacked vertebrates with a thick indumentum, ruling out a dispersal function for *Cardiocarpus* sp. spines. Instead, it is evident that these spines acted as a herbivory defence, likely preventing insect landings, movement and ovipositor insertion for egg deposition into the soft and nutritious seed tissues. The presence of hairs would also impede insects with sucking or chewing mouthparts from accessing the inner parts of the seeds, acting as a deterrent physical barrier.

In modern seeds, the seed coat also provides a physical (and, in some cases, chemical) defence against herbivores. Although effective against many generalist consumers, a thick integument can be overcome by insects with ovipositors or mouthparts specialized for penetrating seed coats [8]. The Shanxi Permian assemblages contain some seeds with a moderately thick and apparently robust seed coat (Fig 6D) that may have acted as a barrier to generalist herbivores [8]. We also note that a range of seeds from other Permian floras are characterised by thick-walled seeds [28, 37, 54, 64–67], suggesting widespread investment in seed protection by plants.

The early Permian of Cathaysia hosted insect communities hostile to seeds (as denoted by the presence of various seed herbivory patterns) but certain gymnosperms were already defending their vulnerable reproductive structures with a diverse array of anti-herbivore physical defences. We cannot determine from the fossils whether any chemical defences were employed by these plants. The defences seem to have been effective against generalist and, potentially, specialist insects as all the damage was found in relatively defenceless seeds despite seeds with potential physical defences being more abundant in these floral assemblages (74 seeds with some type of potential defence).

### 5.3. Putative culprits and paleoecological insights

Identifying potential perpetrators of seed damage in the fossil record is difficult, and it is especially challenging for Paleozoic biotas in which entomofaunas were dominated by extinct insect groups (e.g., Paolidae, Megasecoptera, or some members of Palaeodyctioptera, Archaeorthoptera or early fossil roachoids) with unresolved feeding and egg-laying strategies. In modern ecosystems, a diverse array of species across six prominent insect orders—Coleoptera, Hemiptera, Hymenoptera, Thysanoptera, Diptera and Lepidoptera—are known for their seed-feeding habits [8, 12, 30, 31], but these groups were scarce or absent during the early Permian.

Based on the size and morphological differences in the damage features identified in the Shanxi seed assemblages, it is apparent that several types of insects were responsible for the herbivory. DT430, evident in *Carpolithus bullatus*, appears to have been inflicted by a long and robust ovipositor (see discussion details in the Results section). This ovipositor seems to have been capable of penetrating the resistant integument to reach the nutritive inner tissues of the ovule/seed, resulting in a distinctive and pronounced pattern of oviposition. Evidently, the culprit of this damage type belonged to a group of insects with well-developed ovipositors. Insects with the earliest known ovipositors developed in the late Paleozoic [68–72], and some groups with strong ovipositors (including Palaeodictyopteroidea, Dictyoptera, Odonatoptera, Archaeorthoptera, Hemipteroidea and some early holometabolan insects) have been claimed as putative culprits of endophytic oviposition in other plant-tissues during this interval [69, 71, 73–76].

**Fig 7:**
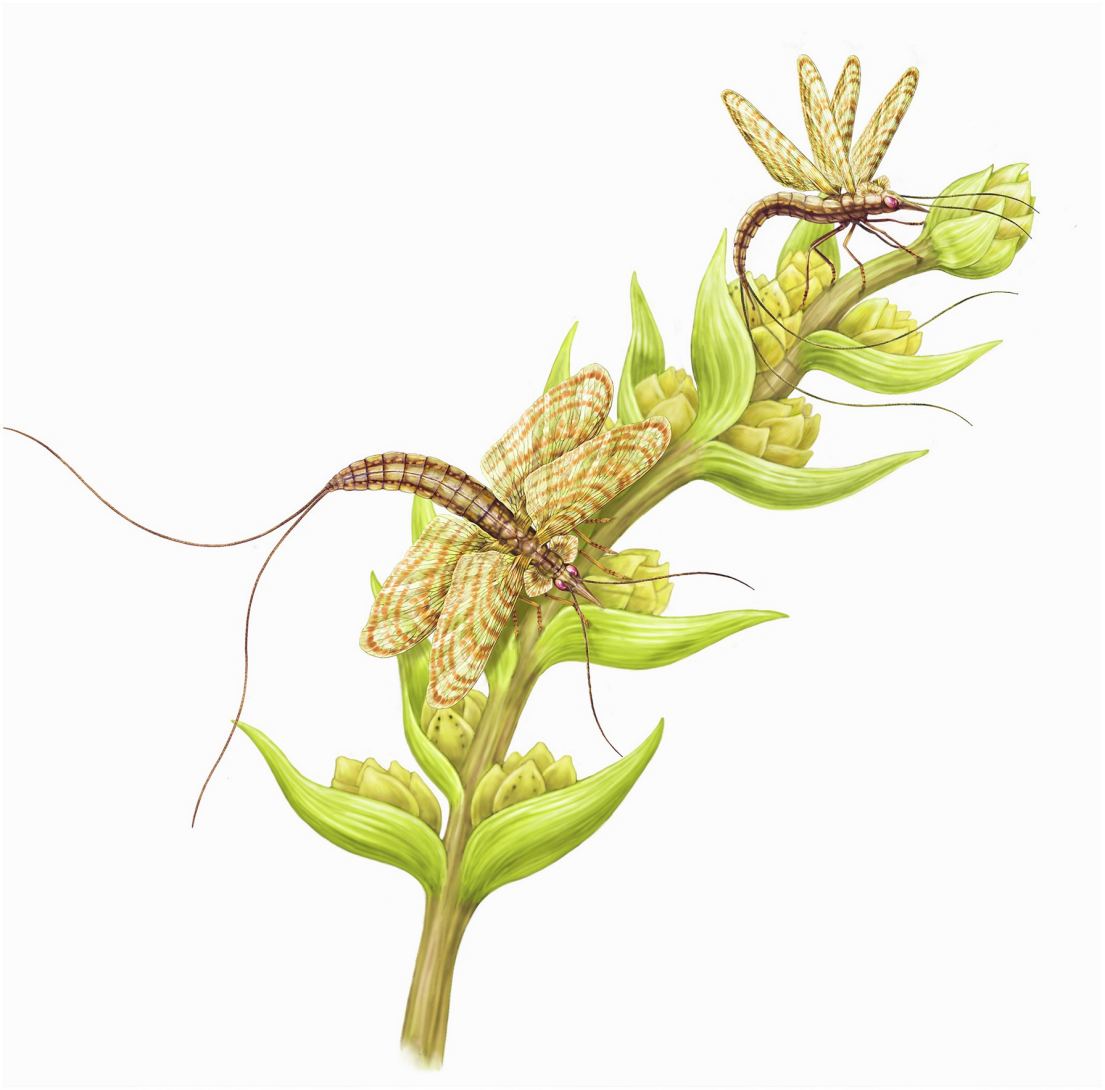
Reconstruction of two palaeodyctiopteran insects laying their eggs in the seeds arranged in a Permian gymnosperm cone (cf. cordaitalean) bearing seeds compatible with *Carpolithus bullatus*; Paleoartist: Pollyanna von Knorring (NRM).

The disparity in size between some oviposition scars in the Shanxi seeds is notably striking. Although taphonomic and preservational factors might have caused some size distortion, certain scars have unequivocally large dimensions, reaching an average of 3.8 x 3 mm in the new DT430 (*Carpolithus bullatus*). These large dimensions accord with the proposition by some authors [76] that certain late Paleozoic insect groups with large ovipositors were potentially responsible for laying larger eggs, although these groups (e.g., palaeodictyopteroids) experienced a decline during the Pennsylvanian–Permian transition. Endophytic oviposition scars even larger than those on *C*. *bullatus* have been found on other Paleozoic fossils [77]. Other authors have suggested Rostropalaeoptera (Paleodictyoptera) as more likely candidates for endophytic egg laying in the late Paleozoic [78]. Based on all of these factors, we cannot exclude Dictyoptera, Odonatoptera, Archaeorthoptera, Hemipteroidea, or early holometabolan insects as culprits of DT430. Nevertheless, a representative of Palaeodictyopteroidea (Fig 7) seems to be the most likely culprit because of the strong and large ovipositors in some species, and the known presence of this group in the Shanxi Formation [79, 80].

Seed predation appears to have been moderately common (three DTs affecting the 7% of the seed assemblage) in the early Permian lowland forests of Cathaysia. Granivory significantly influences seed viability and, thus, seedling establishment. It plays a pivotal role in determining plant abundance, diversity and coexistence within terrestrial ecosystems [1–4]. Based on the results of this study and a growing body of evidence from other regions around the world (see Table 1), it seems that this functional feeding group played a key role in many late Paleozoic ecosystems involving both insects and plants. This is reinforced by evidence of physical plant-defences against seed predation in various seeds.

### 5.4. Comparison with other evidence of Paleozoic seed-predation

**Fig 8:**
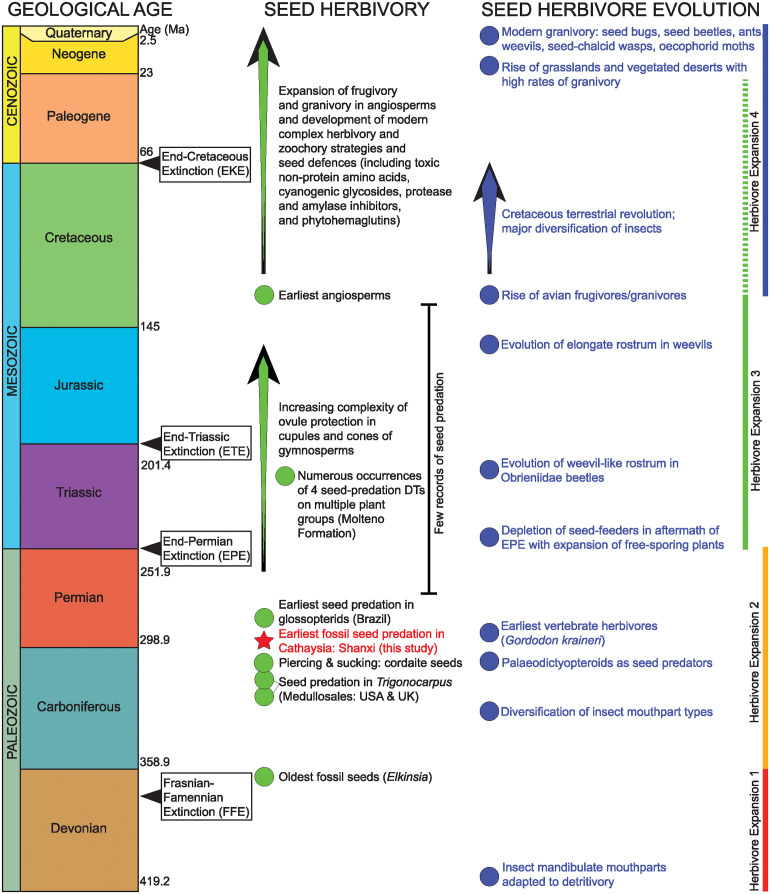
Key events in the evolution of the granivory (seed predation functional feeding group) through geological time. The star shows the position of the new examples of seed predation studied here, representing the earliest occurrences of granivory in Cathysia.

There are several constraints on comparing the Shanxi seed-predation data with equivalent assemblages from the Paleozoic. One is the sparse evidence of seed predation in Paleozoic assemblages in general (Table 1, see Fig 8). Most records of granivory from this interval have been based on single or very few seeds [20, 21, 26, 28, 29]. Only one study from the Carboniferous or early Permian was based on a greater number of specimens [25]. Secondly, the late Paleozoic covers a long interval of time, and the available granivory records derive from distant paleogeographical regions meaning that many of the studied damage features occur on seeds of disparate plant taxa.

Our work provides the first report of seed-predation from Cathaysia. Laurussian fossil collections have received greater attention regarding granivory compared to other areas. Examples of granivory are known from radiospermic seeds of Medullosales from the upper Carboniferous (Pennsylvanian) and lower Permian (Cisuralian), and on platyspermic seeds linked to Cordaitales [19–24, 27]. The Gondwanan flora hosts even fewer records of seed predation, represented primarily by early Permian seeds linked to cordaitalean and glossopteridalean species from Brazil, South Africa and potentially India [25, 26]. Owing to their widespread occurrence, yet few reported examples, considerable research is needed to elucidate the origins and early development of this functional feeding group.

## 6. Conclusions

This study highlights the intricate dynamics of seed predation and anti-herbivore defences during the early Permian in Cathaysia. Three types of damage, including two new types, expand the known diversity of the seed predation functional feeding group. We relate these to different strategies of feeding and reproduction, as oviposition and consumption in Paleozoic seeds. We identify distinct damage patterns, such as DT430, which were probably inflicted by insects with well-developed ovipositors, potentially belonging to Palaeodictyopteroidea.

The relatively common occurrence of seed predation in the early Permian tropical forests of Cathaysia (about 7% of seeds damaged) highlights the ecological significance of granivory in shaping plant abundance and diversity, but also the importance of this feeding strategy for the Permian entomofaunas of this region. Additionally, the development of anti-herbivore defences, ranging from chemical compounds to physical structures, is traced back to the Devonian, with a notable gap in research on Paleozoic seeds. The Shanxi seeds, however, provide evidence of diverse anti-herbivory strategies, including hairs, spines, apical horns and thick seed coats, highlighting the early involvement of seed-defences and seed-predators in the plant-insect evolutionary arms race.

Comparisons with other Paleozoic examples of seed predation reveal the need for continued research to elucidate the sparse fossil record of this herbivory style. This study from Cathaysia adds a crucial piece to the puzzle, expanding our knowledge of global seed-predation dynamics in the late Paleozoic tropical ecosystems.

## References

[1] Wright SJ, Duber HC. Poachers and forest fragmentation alter seed dispersal, seed survival, and seedling recruitment in the palm *Attalea butyraceae*, with implications for tropical tree diversity 1. Biotropica. 2001;33(4):583–595.

[2] Dracxler CM, Pires AS, Fernandez FA. Invertebrate seed predators are not all the same: seed predation by bruchine and scolytine beetles affects palm recruitment in different ways. Biotropica. 2011;43(1):8–11.

[3] Jia S, Wang X, Yuan Z, Lin F, Ye J, Hao Z, et al. Global signal of top-down control of terrestrial plant communities by herbivores. Proc Natl Acad Sci USA. 2018;115(24):6237–6242. doi: 10.1073/pnas.1707984115 29848630 PMC6004463

[4] Williams PJ, Ong RC, Brodie JF, Luskin MS. Fungi and insects compensate for lost vertebrate seed predation in an experimentally defaunated tropical forest. Nat Commun. 2021;12(1):1650. doi: 10.1038/s41467-021-21978-8 33712621 PMC7955059

[5] Vandermeer JH. A graphical model of insect seed predation. Am Nat. 1975;109(966):147–160.

[6] Bohart GE, Koerber TW. Insects and seed production. In: Kozlowski TT, editor. Insects, and Seed Collection, Storage, Testing, and Certification. Ney York, Academic Press; 1972: p. 1–53.

[7] Sarmiento C, Zalamea PC, Dalling JW, Davis AS, Stump SM, et al. Soilborne fungi have host affinity and host-specific effects on seed germination and survival in a lowland tropical forest. Proc Natl Acad Sci USA. 2017;114(43):11458–11463. doi: 10.1073/pnas.1706324114 28973927 PMC5664508

[8] Janzen DH. Seed predation by animals. Annu Rev Ecol Syst. 1971;2(1):465–492.

[9] Siemens DH, Johnson CD, Ribardo KJ. Alternative seed defense mechanisms in congeneric plants. Ecology. 1992;73:2152–2166.

[10] Sercu BK, Moeneclaey I, Bonte D, Baeten L. Induced phenological avoidance: a neglected defense mechanism against seed predation in plants. Journal of Ecology. 2020;108(3):1115–1124.

[11] Moreira X, Abdala-Roberts L, Bruun HH, Covelo F, De Frenne P, Galmán A et al. Latitudinal variation in seed predation correlates with latitudinal variation in seed defensive and nutritional traits in a widespread oak species. Ann Bot. 2020;125(6):881–890. doi: 10.1093/aob/mcz207 31858135 PMC7218813

[12] Moreira X, Pérez-Ramos IM, Matías L, Francisco M, García-González A, Martins-Noguerol R et al. Effects of soil abiotic factors and plant chemical defences on seed predation on sea fennel (*Crithmum maritimum*). Plant Soil. 2021;465(1–2):289–300.

[13] McCoy VE, Wappler T, Labandeira CC. Exceptional fossilization of ecological interactions: plant defenses during the four major expansions of arthropod herbivory in the fossil record. In: Gee CT, McCoy VE, Sander M, editors. Fossilization: Understanding the Material Nature of Ancient Plants and Animals. Baltimore, MD: Johns Hopkins Univ. Press; 2021. p. 187–220.

[14] Howe HF, Westley LC. Ecological relationships of plants and animals. Oxford: University Press; 1988.

[15] Sallabanks R, Courtney SP. Frugivory, seed predation, and insect-vertebrate interactions. Annu Rev Entomol. 1992;37(1):377–400. doi: 10.1146/annurev.en.37.010192.002113 1539939

[16] Hesler LR, Whetzel HH. Manual of Fruit Diseases. New York: MacMillan; 1917.

[17] Labandeira CC, Wappler T. Arthropod and pathogen damage on fossil and modern plants: Exploring the origins and evolution of herbivory on land. Annu Rev Entomol. 2023;68:341–361. doi: 10.1146/annurev-ento-120120-102849 36689301

[18] Buatois LA, Davies NS, Gibling MR, Krapovickas V, Labandeira CC, MacNaughton RB., et al. The Invasion of the Land in Deep Time: Integrating Paleozoic Records of Paleobiology, Ichnology, Sedimentology, and Geomorphology. Integr Comp Biol. 2022;62(2):297–331. doi: 10.1093/icb/icac059 35640908

[19] Sharov AG., Morphological features and mode of life of the Palaeodictyoptera. In: Bei–Benko GY, editor. Readings in the Memory of Nicolaj Aleksandrovich Kholodovskij. Leningrad: Academy of Sciences; 1973. p. 49–63. Russian.

[20] Jennings JR. Lower Pennsylvanian plants of Illinois I: a flora from the Pounds Sandstone Member of the Caseyville Formation. J Paleontol. 1974;48:459–473.

[21] Scott AC, Taylor TN. Plant/animal interactions during the Upper Carboniferous. Bot Rev. 1983;49:259–307.

[22] Shcherbakov DE. On Permian and Triassic insect faunas in relation to biogeography and the Permian–Triassic crisis. Paleont J. 2008;42:15–31.

[23] Schachat SR, Labandeira CC, Chaney DS. Insect herbivory from early Permian Mitchell Creek Flats of north-central Texas: opportunism in a balanced component community. Palaeogeogr Palaeoclimatol Palaeoecol. 2015;440:830–847.

[24] Labandeira CC, Kustatscher E, Wappler T. Floral assemblages and patterns of insect herbivory during the Permian to Triassic of Northeastern Italy. PLoS One. 2016;11(11):e0165205. doi: 10.1371/journal.pone.0165205 27829032 PMC5102457

[25] Barbosa dos Santos T, De Souza Pinheiro ER, Iannuzzi R. First evidence of seed predation by arthropods from Gondwana and its early Paleozoic history (Rio Bonito Formation, Paraná Basin, Brazil). Palaios. 2020;35:292–301.

[26] McLoughlin S, Prevec R, Slater BJ. Arthropod interactions with the Permian Glossopteris flora. J Palaeosciences. 2021;70:43–133.

[27] Schachat SR, Labandeira CC, Gordon J, Chaney D, Levi S, Halthore MN, et al. Plant-insect interactions from early Permian (Kungurian) Colwell Creek Pond, north-central Texas: the early spread of herbivory in riparian environments. Int J Plant Sci. 2014;175(8):855–890.

[28] Anderson JM, Anderson HM. Palaeoflora of southern Africa. Prodromus of South African megafloras Devonian to Lower Cretaceous. Rotterdam: A.A. Balkema; 1985.

[29] Chandra S, Singh KJ. Plant fossils from the type locality of Talchir Formation and evidence of earliest plant–animal activity in Gondwana of India. In: Ayyasami K, Sengupta S, Ghosh RN, editors. Gondwana Nine. Rotterdam: A.A. Balkema; 1996. p. 397–414.

[30] Lewis OT, Gripenberg S. Insect seed predators and environmental change. J Appl Ecol. 2008;45(6):1593–1599.

[31] Roques A, Copeland RS, Soldati L, Denux O, Auger-Rozenberg MA. *Megastigmus* seed chalcids (Hymenoptera, Torymidae) radiated much more on Angiosperms than previously considered. I-Description of 8 new species from Kenya, with a key to the females of Eastern and Southern Africa. Zookeys. 2016;585:51–124. 10.3897/zookeys.585.7503 PMC4857038 27199604

[32] Halle TG. Palaeozoic plants from Central Shansi. Pal Sin, Ser A. 1927;2:1–316.

[33] Shen BH, Shen SZ, Wu Q, Zhang SC, Zhang B, Wang XD, et al. Carboniferous and Permian integrative stratigraphy and timescale of North China Block. Sci China Earth Sci. 2022;65:983–1011.

[34] Wu Q, Ramezani J, Zhang H, Wang J, Zeng F, Zhang Y, et al. High-precision U-Pb age constraints on the Permian floral turnovers, paleoclimate change, and tectonics of the North China block. Geology. 2021;49(6):677–681.

[35] Stevens LG, Hilton J, Bond DPG, Glasspool IJ, Jardine PE. Radiation and extinction patterns in Permian floras from North China as indicators for environmental and climate change. J Geol Soc London. 2011;168:607–619.

[36] Kong X, Xu X, Chang JL, Liu LJ, Zhao XH, Zhang LX. Late Palaeozoic coal-bearing strata and biota in Shanxi, China. Taiyuan: Shanxi Science and Technology Press; 1996. Chinese with English summary.

[37] Hilton J, Rothwell GW, Li C-S, Wang S-J, Galtier J. Permineralised cardiocarpalean ovules in wetland vegetation. Palaeontology. 2001;44:811–825.

[38] Hilton J, Cleal CJ. The relationship between Euramerican and Cathaysian tropical floras in the Late Palaeozoic: palaeobiogeographical and palaeogeographical implications. Earth Sci Rev. 2007;85:85–116.

[39] Geng B, Hilton J. New coniferophyte ovulate structures from the Early Permian of China. Bot J Linn Soc. 1999;129:115–138.

[40] Wang ZQ. Palaeovegetation and plate tectonics: phytogeography of North China during Permian and Triassic times. Palaeogeogr Palaeoclimatol Palaeoecol. 1985;49:25–45.

[41] Wang ZQ. Permian gigantic palaeobotanic events in North China. Acta Palaeontol Chin. 1989;28:314–343.

[42] Norin E. The late Palaeozoic and early Mesozoic sediments of central Shansi. Bull Geol Sur China. 1922;4:1–79.

[43] Norin E. The lithological character of the Permian sediments of the Angara Series in central Shansi, N. China. GFF. 1924;46:19–55.

[44] Yang JH, Cawood PA, Du YS, Feng B, Yan JX. Global continental weathering trends across the Early Permian glacial to postglacial transition: Correlating high- and low-paleolatitude sedimentary records. Geology. 2014;42:835–838.

[45] Yang JH, Wu FY, Shao JA, Wilde SA, Xie LW, Liu XM. Constraints on the timing of uplift of the Yanshan Fold and Thrust Belt, North China. Earth Planet Sci Lett. 2006;246:336–352.

[46] Sun JP, Yang L, Dong Y P, Yang XY, Peng Y, Zhao J. Permian tectonic evolution of the southwestern Ordos Basin, North China: Integrating constraints from sandstone petrology and detrital zircon geochronology. Geol J. 2020;55:8068–8091.

[47] Luo SS, Pan ZY, Lv QQ, He WL, Wen S. The Upper Paleozoic detrital zircon U-Pb geochronology and its tectonic significance in southwestern Ordos Basin. Geol China. 2017;44:556–574. Chinese.

[48] Zhu XQ, Zhu WB, Ge RF, Wang X. Late Paleozoic provenance shift in the south-central North China Craton: Implications for tectonic evolution and crustal growth. Gondwana Res. 2014;25:383–400.

[49] Liu GH. Permo-Carboniferous palaeogeography and coal accumulation and their tectonic control in the North and South China continental plates. Int J Coal Geol. 1990;16:73–117.

[50] Zhang D, Huang B., Zhao G, Meert JG, Williams S, Zhao J, et al. Quantifying the Extent of the Paleo-Asian Ocean During the Late Carboniferous to Early Permian. Geophys Res Lett. 2021;48:e2021GL094498.

[51] Wang Q. (1996) Proposal to conserve the name *Carpolithus* with that spelling (fossil Spermatopsida). Taxon. 2011;60:241–242.

[52] Cleal CJ, Thomas BA. Nomenclatural Status Of The Palaeobotanical “Artificial Taxa” Established In Brongniart’s 1822 “Classification” Paper. Foss Imp. 2018;74:9–28.

[53] Hilton J, Wang S-J, Zhu W-Q, Tian B, Galtier J, Wei A-H. Callospermarion ovules from the early Permian of northern China: palaeofloristic and palaeogeographic significance of callistophytalean seed-ferns in the Cathaysian flora. Rev Palaeobot Palynol. 2002;120:301–314.

[54] Labandeira CC, Wilf P, Johnson KR, Marsh F. Guide to insect (and other) damage types on compressed plant fossils. Washington DC: Smithsonian Institution, National Museum of Natural History, Department of Paleobiology; 2007.

[55] Šimůnek Z, Cleal CJ. Imparipinnate neuropteroid foliage (Medullosales) from the middle Westphalian of the west and Central Bohemia Coal Basin, Czech Republic. Rev Palaeobot Palynol. 2011;166(3–4):163–201.

[56] Gillespie WH, Pfefferkorn HW. Taeniopterid lamina on *Phasmatocycas* megasporophylls (Cycadales) from the Lower Permian of Kansas, U.S.A. Rev Palaeobot Palynol. 1986;49:99–116.

[57] Wang S-J, Bateman RM, Spencer ART, Wang J, Shao L, Hilton J. Anatomically preserved “strobili” and leaves from the Permian of China (Dorsalistachyaceae, fam. nov.) broaden knowledge of Noeggerathiales and constrain their possible taxonomic affinities. Am J Bot 2017;104:127–149. doi: 10.3732/ajb.1600371 28062406

[58] Seyfullah LJ, Hilton J. Re–evaluation of Halle’s fertile pteridosperms from the Permian floras of Shanxi Province, China. Plant Syst Evol. 2009;279:191–218.

[59] Fürstenberg-Hägg J, Zagrobelny M, Bak S. Plant defense against insect herbivores. Int J Mol Sci. 2013;14(5):10242–10297. doi: 10.3390/ijms140510242 23681010 PMC3676838

[60] Colston CM, Landaw K, Tomescu AM. An early snapshot of plant–herbivore interactions: *Psilophyton diakanthon* sp. nov. from the Early Devonian of Gaspé (Quebec, Canada). Am J Bot. 2023;110(1):e16082. 36219504 10.1002/ajb2.16082

[61] Krings M, Taylor TN, Kellogg DW. Touch-sensitive glandular trichomes: a mode of defence against herbivorous arthropods in the Carboniferous. Evol Ecol Res. 2002;4:779–786.

[62] Krings M, Kellogg DW, Kerp H, Taylor TN. Trichomes of the seed fern *Blanzyopteris praedentata*: implications for plant–insect interactions in the Late Carboniferous. Bot J Linn Soc. 2003;141(2):133–149.

[63] McLoughlin S, Pott C. Plant mobility in the Mesozoic: disseminule dispersal strategies of Chinese and Australian Middle Jurassic to Early Cretaceous plants. Palaeogeogr Palaeoclimatol Palaeoecol. 2019;515:47–69.

[64] Taylor TN. Observations on *Stephanospermum* ovoides, a Middle Pennsylvanian Seed. Am J Bot. 1962;49:794–800.

[65] McLoughlin S, Bomfleur B. Drinnan AN. *Pachytestopsis tayloriorum* gen. et sp. nov., an anatomically preserved glossopterid seed from the Lopingian of Queensland, Australia. Krings M, Harper CJ, Cúneo NR, Rothwell GW, editors. Transformative Paleobotany: Papers to Commemorate the Life and Legacy of Thomas N. Taylor. Amsterdam: Elsevier; 2018. p. 155–178.

[66] McLoughlin S, Maksimenko A, Mays C. A new high-paleolatitude permineralized peat flora from the late Permian of the Sydney Basin, Australia. International Journal of Plant Sciences. 2019;180:513–539.

[67] Prevec R. *Elatra*: a glossopterid fructification with a bipartite, hooded wing from the lower Permian of Madagascar and South Africa. Rev Palaeobot Palynol. 2014;210:119–139.

[68] Zeh DW, Zeh JA, Smith RL. Ovipositors, amnions and eggshell architecture in the diversification of terrestrial arthropods. Q Rev Biol. 1989;64:147–168.

[69] Labandeira CC. Silurian to Triassic plant and hexapod clades and their associations: New data, a review, and interpretations. Arthropod Syst Phylogeny. 2006;64:53–94.

[70] Laaß M, Hoff C. The earliest evidence of damselfly-like endophytic oviposition in the fossil record. Lethaia. 2015;48(1):115–124.

[71] Santos AA, Hernández-Orúe A, Wappler T, Diez JB. Plant–insect interactions from the Late Pennsylvanian of the Iberian Peninsula (León, northern Spain). Rev Palaeobot Palynol. 2022;301:104658.

[72] Santos AA., Hernández-Orúe A, Wappler T, Peñalver-Mollá E, Diez JB, Nel A. Late Carboniferous insects from the Iberian Peninsula: state of the art and new taxa. Palaeontographica Abt A. 2023;326:1–27.

[73] Grimaldi D, Engel MS. The Evolution of the Insects. Cambridge: Cambridge University Press; 2005.

[74] Hornig MK, Haug C, Schneider JW, Haug JT. Evolution of reproductive strategies in dictyopteran insects-clues from ovipositor morphology of extinct roachoids. Acta Palaeontol Pol. 2018;63(1):1–24

[75] Laaß M, Hauschke N. Earliest record of exophytic insect oviposition on plant material from the latest Pennsylvanian (Gzhelian, Stephanian C) of the Saale Basin, Germany. Palaeogeogr Palaeoclimatol Palaeoecol. 2019;534:109337.

[76] Romero-Lebrón E, Robledo JM, Delclòs X, Petrulevičius JF, Gleiser RM. Endophytic insect oviposition traces in deep time. Palaeogeogr Palaeoclimatol Palaeoecol. 2022;590:110855.

[77] Béthoux O, Galtier J, Nel A. Earliest evidence of insect endophytic oviposition. Palaios. 2004;19(4):408–413.

[78] Chen L, Gu JJ, Yang Q, Ren D, Blanke A, Béthoux O. Ovipositor and mouthparts in a fossil insect support a novel ecological role for early orthopterans in 300 million years old forests. eLife. 2021;10:e71006. doi: 10.7554/eLife.71006 34844668 PMC8631945

[79] Hong YC. New fossil genera and species of Shanxi Formation in Xishan of Taiyuan. Entomotaxonomia. 1985;7(2):83–91. Chinese.

[80] Hong Y. Establishment of fossil entomofaunas and their evolutionary succession in North China. Insect Sci. 1998;5(4):283–300.

